# Supplementary material for: Parallelised detection of bacteria viability using an electrode array and the Exeter Multiscope
Source: Sci Rep. 2026 Aug 25;16:29903. doi: 10.1038/s41598-026-66381-9 (PMC13614890; doi:10.1038/s41598-026-66381-9)
Supplement: Supplementary file 1 [file 41598_2026_66381_MOESM1_ESM.docx]

Supplementary information for:

“Parallelised detection of bacteria viability using an electrode array and the Exeter Multiscope”.

Outlined below are the standard operating procedures (SOPs) used for the acquisition of data described in the main text. These include:

- CytePulse imaging on the Multiscope
- CytePulse imaging on the Inverted Microscope (Olympus IX73)

**CytePulse Imaging on the Multiscope**

| **SOP WRITTEN BY:** | **KA KIU LEE** |
| --- | --- |
| **AUTHORISED BY:** | **Alex Corbett** |
| **VERSION No:** | **01** |
| **EFFECTIVE DATE:** | 16 July 2025 |
| **REVIEW DATE:** |  |

**Contents:**

1. Culturing bacteria
2. CyteCom stock solution preparation for agarose pads
3. CyteCom agarose pad preparation
4. Preparation of agarose pads for bacterial spotting and CytePulse electrode mounting
5. CytePulse device operation
6. Imaging on the Multiscope with Thonny and Micro-Manager 2.0 Software
7. CytePulse electrode cleaning protocol
8. Image analysis on Fiji ImageJ

**Culturing bacteria:**

**Streak plate:**

1. Prepare streak plate (LB 1.5% agar with 10 g/L NaCl) with bacteria from cryostock.
2. Incubate at 37 °C for 17 h.
3. Store at 4 °C for up to 14 days.

**Liquid culture:**

1. Inoculate 100 mL fresh of 10 g/L NaCl LB in a 250 mL conical flask by transferring a single colony from streak plate.
2. Incubate at 37 °C and 200 rpm for 2-3 h to reach log phase bacteria (OD_600_ ~1.5) for *Escherichia coli* BW25113, *Bacillus subtilis* 168, *Pseudomonas aeruginosa* PA14, *Staphylococcus aureus* MSSA476.

**CyteCom Stock Solution Preparation for Agarose Pads:**

**Base:**

| **3-(N-Morpholino)propanesulfunoic acid (MOPS) (1 M)** | **MW (g/mol)** | **For 500 mL (g)** |
| --- | --- | --- |
| - MOPS Buffer | 209.26 | 20.926 |
| - Sodium Hydroxide Pellets for pH 7 |  | ≥3 pellets |

1. Add the specified weight of MOPS to a clean 500 mL Duran bottle.
2. Add 400 mL of distilled water to bottle.
3. Stir in sodium hydroxide pellets (~3) to the MOPS solution until pH 7 is reached using a magnetic stirrer. Measure pH using a pH probe or indicator strips.
4. Top up to 500 mL with distilled water.
5. Label with autoclave tape indicating molarity and date, then autoclave.

| **Potassium Phosphate buffer (0.5 M)** | **MW (g/mol)** | **For 500 mL (g)** |
| --- | --- | --- |
| - Potassium Phosphate Monobasic | 136.09 | 16.609 |
| - Potassium Phosphate Dibasic | 174.18 | 21.046 |

1. Add the specified weights of potassium phosphate monobasic and dibasic to a clean 500 mL Duran bottle.
2. Ensure the pH is 7 with indicator strips.
3. Label with autoclave tape indicating molarity and date, then autoclave.

**Carbon/nitrogen source with trace ions:**

| **Trace Ion Solutions** | **Required Molarity or %** | **For 10 mL** |
| --- | --- | --- |
| L-glutamate | 25% w/v | 2.5 g |
| Glycerol | 50% v/v | 5 mL |
| Glucose | 25% w/v | 2.5 g |
| Sodium Chloride (NaCl) | 25% w/v | 2.5 g |
| Ammonium Chloride (NH_4_Cl) | 0.1 M | 0.0534 g |
| Calcium Chloride (CaCl_2_) | 0.7 M | 0.7769 g |
| Magnesium Chloride (MgCl_2_) | 2 M | 1.9042 g |
| Iron(III) Chloride (FeCl_3_) | 0.1 M | 0.1622 g |
| Manganese(II) Chloride (MnCl_2_) | 0.05 M | 0.06292 g |
| Thiamine | 0.1 M | 0.33727 g |
| Zinc(II) Chloride (ZnCl_2_) | 0.1 M | 0.1363 g |
| Thioflavin T (ThT) | 10 mM | 0.0318 g |

1. Add the specified weight of the compound to 10 mL of distilled water into a sterile tube.
2. Remove the 10 mL solution using a sterile syringe and filter using a 0.2 μm syringe filter into a new sterile tube.
3. Label tube with compounds name, molarity or %, and date then store in the fridge.
4. Do not autoclave any of these compounds.

**CyteCom Agarose Pad Preparation:**

**Recipe for a total of 20 mL molten agarose (adjust volumes as necessary):**

**Base:**

| Milli-Q Water | 20 mL |
| --- | --- |
| 3-(N-Morpholino)propanesulfunoic acid (MOPS) (use near a Bunsen burner) | 200 μL |
| Potassium Phosphate buffer 0.5M (use near a Bunsen burner) | 200 μL |
| UltraPure Agarose (if solid required) | 0.4 g |

**Autoclave or Microwave to Dissolve Agarose and place in 60 °C water bath**

**Carbon/nitrogen source with trace ions:**

| L-glutamate (25% w/v) (prepare new stock every 2 weeks) | 400 μL |
| --- | --- |
| Glycerol (50% v/v) | 200 μL |
| Glucose (25% w/v) | 200 μL |
| Sodium Chloride (NaCl) (25% w/v) | 200 μL |
| Ammonium Chloride (NH_4_Cl) (0.1 M) | 200 μL |
| Calcium Chloride (CaCl_2_) (0.7 M) | 20 μL |
| Magnesium Chloride (MgCl_2_) (2 M) | 20 μL |
| Iron(III) Chloride (FeCl_3_) (0.1 M) | 20 μL |
| Manganese(II) Chloride (MnCl_2_) (0.05 M) | 20 μL |
| Thiamine (0.1 M) | 0.4 μL |
| Zinc(II) Chloride (ZnCl_2_) (0.2 M) | 0.2 μL |

**Thioflavin T (ThT) (10 mM):**

| Gram Positive Species | 64 μL |
| --- | --- |
| Gram Negative Species | 128 μL |

**Preparation of Agarose Pads for Bacterial Spotting and CytePulse Electrode Mounting:**

1. Prepare agarose pads next to Bunsen burner to maintain aseptic conditions.
2. Prepare bench surface by casting parafilm on the surface (hydrophobic parafilm will help immobilise glass coverslip when handling the agarose pad).
3. Place a 22 × 22 mm coverslip onto the parafilm surface for transfer of molten agarose.
4. Pipette 750 μL to 1,000 μL of the molten agarose onto each coverslip.
5. Gently place a second 22 × 22 mm coverslip on top of the molten agarose to create an “agarose sandwich”.
6. Cover agarose pads with a petri dish and allow to solidify (~10 min).
7. Remove top coverslip by sliding off the solidified agarose and dispose coverslip in a sharps bin.
8. Using a sterile scalpel, cut up each 22 × 22 mm agarose pad into 16 equal sections (4 × 4 grid).
9. Allow agarose pad to dry off so that no excess liquid is on the agarose pad surface.
10. Add 1-2 μL of log phase bacteria onto each agarose pad section as required and allow to adsorb into the pad.
11. Using a sterile scalpel, mount the agarose pad section onto the CytePulse electrode by flipping it so that the bacterial side if facing down onto the electrode. Ensure the agarose pad is not touching the ground electrode in the centre of the device.
12. Place onto the custom 3D-printed electrode mount shown below. Connect IDC cable and then place chimney mount on top to reduce cross-talk.

**CytePulse Device Operation:**

1. Ensure the CytePulse device is charged using the USB-C charger.
2. Connect CytePulse device to electrode using an IDC cable.

3. Turn on the device with the silver power button on the left hand side.
4. Connect a keyboard to the USB-A port.
5. Log in using to the “CyteCountAdmin” account using the password provided.
6. Open the Arduino Software
7. Load the script named “CytePulse_modified_VoltageRange”
8. Open the command console by clicking the magnifying glass icon in the top right corner of the Arduino interface. This will open the “COM3” window, where you can enter commands as shown in the Command reference table below.
9. Set the parameters using the Command reference parameters and then use the Run command to conduct the experiments.
10. Close Arduino software when experiment is complete.
11. Shut down CytePulse device by pressing on the Windows icon on the bottom left corner > “Power” > “Shut down”.

**Command reference:**

| **Parameter** | **Description** | **Input** | **Example** |
| --- | --- | --- | --- |
| Voltage | Sets the voltage rails. Both negative and positive rails must be configured separately | set negativeRail (p) (n) | set negativeRail 1 160 |
|  |  | set positiveRail (p) (n) | set positiveRail 1 0.7 |
| Frequency | Sets the output frequency in Hz | set frequency (p) (n) | set frequency 1 100 |
| Verify | Verifies current settings before delivering a pulse. | run verify (p) | run verify 1 |
| Run | Delivers the pulse (shock) to the specified position. | run start (p) | run start 1 |

p = Electrode position

n = Desired numerical setting (e.g. frequency in Hz)

**Voltage Lookup Table:**

| **Voltage (V)** | **negativeRail** | **positiveRail** |
| --- | --- | --- |
| 3.50 | 170 | 0.6 |
| 3.75 | 165 | 0.65 |
| 4.00 | 160 | 0.7 |
| 4.25 | 157 | 0.75 |
| 4.40 | 152 | 0.78 |
| 4.50 | 145 | 0.8 |
| 4.70 | 135 | 0.8 |
| 5.00 | 125 | 0.82 |
| 5.25 | 100 | 0.84 |

**Imaging on the Multiscope with Thonny and Micro-Manager 2.0 Software:**

**Multiscope:**

1. Turn on camera by flicking switch on the back of the ORCA-Flash4.0 V3.
2. Turn on LED array with the Tenma power supply by pressing the power button, then the ‘On/Off’ button
   a. LED brightness can be adjusted by changing the voltage by pressing the ‘Voltage/Current’ button and twisting the knob to adjust values and the arrow button to select the number position.
   b. **The LEDs are rated to have a maximum voltage of 5.8 V, so do not go over this value.**
3. The LED array is controlled using a custom Thonny script. This script can be found by following this [link](https://universityofexeteruk.sharepoint.com/:u:/r/sites/CorbettLab/Shared%20Documents/Data/MRC%20IAA%20(CyteCom)/Protocols/Multiplexer_double_vTiny_3x3.py?csf=1&web=1&e=TfUhFR).

**Thonny software for LED control:**

| Command | Description | Example |
| --- | --- | --- |
| on() | To switch on individual LED | on(‘e7’) |
| cycle() | To cycle through LEDs. Rate of switch can be configured in the script under:   - def cycle(duration=[enter time in ms).   Script will have to be stopped (Ctrl + C) and restarted for changes to be implemented. | cycle() |
| trigcycle() | To cycle through LEDs and trigger image capture. | trigcycle() |

4 positions on the electrode can be images with the chimney mount:

| **Electrode Position** | **LED Position** |
| --- | --- |
| 1 | F8 |
| 2 | F6 |
| 3 | E8 |
| 4 | E6 |

Positions 4 and 5 can be accessed by removing the chimneys and sliding the electrode mount away from the back whilst using LED positions E6 and F6.

**Imaging and stage control with Micro-Manager 2.0 software:**

1. Open Micro-Manager software.
2. Load hardware configuration and select ‘[ORCA+MCL](https://universityofexeteruk.sharepoint.com/:u:/r/sites/CorbettLab/Shared%20Documents/Data/MRC%20IAA%20(CyteCom)/Protocols/ORCA+MCL.cfg?csf=1&web=1&e=LcUaUD)’.
3. Open stage control by selecting ‘Tools’ and then ‘Stage Control…’.
   a. Adjust increments in the panel. Down arrows move the stage up and up arrows move the stage down.
4. Adjust image settings in ‘Imaging settings’ including exposure and binning.
5. Select ‘Live’ for live view from camera. Deselect and select ‘autostretch’ to adjust the display intensity range of the image in real time to enhance visibility.
6. Save image with the bottom right icon on the live view section.
7. To capture a time series, select ‘ Multi-D Acq.’
   a. Enter the location that you want to save images at the bottom of the Multi-Dimensional Acquisition window, entering the ‘Directory root’, and the ‘Name prefix’ for preferred names. Record electrode position, treatment, and image acquisition details here.
   b. For example, to capture 20 images every 3 s, set the ‘Count’ to 20 and ‘Interval’ to 3,000. The exposure time for each image will have to be under 3 s, i.e. 2,900 ms. Remember to change the exposure to 2900 ms if a different exposure was used for focusing.
   c. Saving format: ‘Separate image files’.
   d. Click ‘Acquire!’.

**CytePulse Electrode Cleaning Protocol:**

For all washing steps, avoid liquid contact with the IDC port and that only the electrode interface between agarose pads are washed.

1. Prior to cleaning the electrode, ensure cleaning baths are visibly clean and have been changed the same day.
2. Remove agarose pads with rubber coated tweezers or two pipette tips. Be careful not to scratch the electrode surface.
3. Bathe the electrode with the stand in 0.1% Sodium Hypochlorite solution for 10 min.
   a. Prepare by diluting thin bleach in MQ water (e.g. 22 mL 4.53% w/w thin bleach in 978 mL MQ water).

4. Rinse with MQ water with a wash bottle.

5. Place the electrode in an ultrasonic cleaner on the stand containing 0.2% w/v ascorbic acid. Bathe for 7 min, then sonicate for 3 min.
   a. Prepare by dissolving 2.0 g ascorbic acid in 1 L MQ water.

6. Bathe the electrode in MQ water for 10 min.
7. Air dry at room temperature electrode side down before storing.

**Image Analysis with Fiji ImageJ:**

1. Open Fiji ImageJ and drag a folder containing the images captured in the kinetic series onto the interface.
2. Click “Yes” when prompted to open the folder as a stack.
3. Adjust the contrast of the stack by selecting a region with bacteria and pressing “Shift + C”, and by selecting “Auto”.
4. Use the function “Plot Z Axis Profile”, found under “Image” > “Stack” > “Plot Z Axis Profile”.
5. A graph of the response of the image stack will be displayed. Press “Live” to update the Z Axis Profile based on the area selected for analysis (i.e. a cluster of cells, or single cells).

**CytePulse Imaging on the Olympus IX73**

| **SOP WRITTEN BY:** | **KA KIU LEE** |
| --- | --- |
| **AUTHORISED BY:** | **Alex Corbett** |
| **VERSION No:** | **01** |
| **EFFECTIVE DATE:** | 15 May 2025 |
| **REVIEW DATE:** |  |

**Contents:**

9. Culturing bacteria
10. CyteCom stock solution preparation for agarose pads
11. CyteCom agarose pad preparation
12. Preparation of Agarose Pads for Bacterial Spotting and CytePulse Electrode Mounting
13. CytePulse Device Operation
14. Imaging on Olympus IX73 Inverted Microscope with Andor Solis Software
15. CytePulse Electrode Cleaning Protocol
16. Image Analysis on Fiji ImageJ

**Culturing bacteria:**

**Streak plate:**

4. Prepare streak plate (LB 1.5% agar with10 g/L NaCl) with bacteria from cyrostock.
5. Incubate at 37 °C for 17 h.
6. Store at 4 °C for up to 14 days.

**Liquid culture:**

3. Inoculate 100 mL fresh of 10 g/L NaCl LB in a 250 mL conical flask by transferring a single colony from streak plate.
4. Incubate at 37 °C and 200 rpm for 2-3 h to reach log phase bacteria (OD_600_ ~1.5) for *Escherichia coli* BW25113, *Bacillus subtilis* 168, *Pseudomonas aeruginosa* PA14, *Staphylococcus aureus* MSSA476.

**CyteCom Stock Solution Preparation for Agarose Pads:**

**Base:**

| **3-(N-Morpholino)propanesulfunoic acid (MOPS) (1 M)** | **MW (g/mol)** | **For 500 mL (g)** |
| --- | --- | --- |
| - MOPS Buffer | 209.26 | 20.926 |
| - Sodium Hydroxide Pellets for pH 7 |  | ≥3 pellets |

6. Add the specified weight of MOPS to a clean 500 mL Duran bottle.
7. Add 400 mL of distilled water to bottle.
8. Stir in sodium hydroxide pellets (~3) to the MOPS solution until pH 7 is reached using a magnetic stirrer. Measure pH using a pH probe or indicator strips.
9. Top up to 500 mL with distilled water.
10. Label with autoclave tape indicating molarity and date, then autoclave.

| **Potassium Phosphate buffer (0.5 M)** | **MW (g/mol)** | **For 500 mL (g)** |
| --- | --- | --- |
| - Potassium Phosphate Monobasic | 136.09 | 16.609 |
| - Potassium Phosphate Dibasic | 174.18 | 21.046 |

4. Add the specified weights of potassium phosphate monobasic and dibasic to a clean 500 mL Duran bottle.
5. Ensure the pH is 7 with indicator strips.
6. Label with autoclave tape indicating molarity and date, then autoclave.

**Carbon/nitrogen source with trace ions:**

| **Trace Ion Solutions** | **Required Molarity or %** | **For 10 mL (g)** |
| --- | --- | --- |
| L-glutamate | 25% w/v | 2.5 g |
| Glycerol | 50% v/v | 5 mL |
| Glucose | 25% w/v | 2.5 g |
| Sodium Chloride (NaCl) | 25% w/v | 2.5 g |
| Ammonium Chloride (NH_4_Cl) | 0.1 M | 0.0534 g |
| Calcium Chloride (CaCl_2_) | 0.7 M | 0.7769 g |
| Magnesium Chloride (MgCl_2_) | 2 M | 1.9042 g |
| Iron(III) Chloride (FeCl_3_) | 0.1 M | 0.1622 g |
| Manganese(II) Chloride (MnCl_2_) | 0.05 M | 0.06292 g |
| Thiamine | 0.1 M | 0.33727 g |
| Zinc(II) Chloride (ZnCl_2_) | 0.1 M | 0.1363 g |
| Thioflavin T (ThT) | 10 mM | 0.0318 g |

5. Add the specified weight of the compound to 10 mL of distilled water into a sterile tube.
6. Remove the 10 mL solution using a sterile syringe and filter using a 0.2 μm syringe filter into a new sterile tube.
7. Label tube with compounds name, molarity or %, and date then store in the fridge.
8. Do not autoclave any of these compounds.

**CyteCom Agarose Pad Preparation:**

**Recipe for a total of 20 mL molten agarose (adjust volumes as necessary):**

**Base:**

| Milli-Q Water | 20 mL |
| --- | --- |
| 3-(N-Morpholino)propanesulfunoic acid (MOPS) (use near a Bunsen burnerd) | 200 μL |
| Potassium Phosphate buffer 0.5M (use near a Bunsen burner) | 200 μL |
| UltraPure Agarose (if solid required) | 0.4 g |

**Autoclave or Microwave to Dissolve Agarose and place in 60 °C water bath**

**Carbon/nitrogen source with trace ions:**

| L-glutamate (25% w/v) (prepare new stock every 2 weeks) | 400 μL |
| --- | --- |
| Glycerol (50% v/v) | 200 μL |
| Glucose (25% w/v) | 200 μL |
| Sodium Chloride (NaCl) (25% w/v) | 200 μL |
| Ammonium Chloride (NH_4_Cl) (0.1 M) | 200 μL |
| Calcium Chloride (CaCl_2_) (0.7 M) | 20 μL |
| Magnesium Chloride (MgCl_2_) (2 M) | 20 μL |
| Iron(III) Chloride (FeCl_3_) (0.1 M) | 20 μL |
| Manganese(II) Chloride (MnCl_2_) (0.05 M) | 20 μL |
| Thiamine (0.1 M) | 0.4 μL |
| Zinc(II) Chloride (ZnCl_2_) (0.2 M) | 0.2 μL |

**Thioflavin T (ThT) (10 mM):**

| Gram Positive Species | 64 μL |
| --- | --- |
| Gram Negative Species | 128 μL |

**Preparation of Agarose Pads for Bacterial Spotting and CytePulse Electrode Mounting:**

13. Prepare agarose pads next to Bunsen burner to maintain aseptic conditions.
14. Prepare bench surface by casting parafilm on the surface (hydrophobic parafilm will to help immobilise glass coverslip when handling the agarose pad).
15. Place a 22 × 22 mm coverslip onto the parafilm surface for transfer of molten agarose.
16. Pipette 750 μL to 1,000 μL of the molten agarose onto each coverslip.
17. Gently place a second 22 × 22 mm coverslip on top of the molten agarose to create an “agarose sandwich”.
18. Cover agarose pads with a petri dish and allow to solidify (~10 min).
19. Remove top coverslip by sliding off the solidified agarose and dispose coverslip in a sharps bin.
20. Using a sterile scalpel, cut up each 22 × 22 mm agarose pad into 16 equal sections (4 × 4 grid).
21. Allow agarose pad to dry off so that no excess liquid is on the agarose pad surface.
22. Add 1-2 μL of log phase bacteria onto each agarose pad section as required and allow to adsorb into the pad.
23. Using a sterile scalpel, mount the agarose pad section onto the CytePulse electrode by flipping it so that the bacterial side if facing down onto the electrode. Ensure the agarose pad is not touching the ground electrode in the centre of the device.
24. Place onto the custom 3D-printed electrode mount that fits into the Olympus IX73 stage plate. Immobilise IDC ribbon cable using the stage clamps.

**CytePulse Device Operation:**

12. Ensure the CytePulse device is charged using the USB-C charger.
13. Connect CytePulse device to electrode using an IDC cable.

14. Turn on the device with the silver power button on the left hand side.
15. Connect a keyboard to the USB-A port.
16. Log in using to the “CyteCountAdmin” account using the password provided.
17. Open the Arduino Software
18. Load the script named “CytePulse_modified_VoltageRange”
19. Upload the script with the arrow button on the top left to default settings (4.0 V and 100 Hz for all 6 positions).
20. Open the command console by clicking the magnifying glass icon in the top right corner of the Arduino interface. This will open the “COM3” window, where you can enter commands as shown in the Command reference table below.
21. Close Arduino software when experiment is complete.
22. Shut down CytePulse device by pressing on the Windows icon on the bottom left corner > “Power” > “Shut down”.
23. Press button, to stop power supply after shut down.

**Command reference:**

| **Parameter** | **Description** | **Input** | **Example** |
| --- | --- | --- | --- |
| Voltage | Sets the voltage rails. Both negative and positive rails must be configured separately | set negativeRail (p) (n) | set negativeRail 1 160 |
|  |  | set positiveRail (p) (n) | set positiveRail 1 0.7 |
| Frequency | Sets the output frequency in Hz | set frequency (p) (n) | set frequency 1 100 |
| Verify | Verifies current settings before delivering a pulse. | run verify (p) | run verify 1 |
| Run | Delivers the pulse (shock) to the specified position. | run start (p) | run start 1 |

p = Electrode position

n = Desired numerical setting (e.g. frequency in Hz)

**Voltage Lookup Table:**

| **Voltage (V)** | **negativeRail** | **positiveRail** |
| --- | --- | --- |
| 3.50 | 170 | 0.6 |
| 3.75 | 165 | 0.65 |
| 4.00 | 160 | 0.7 |
| 4.25 | 157 | 0.75 |
| 4.40 | 152 | 0.78 |
| 4.50 | 145 | 0.8 |
| 4.70 | 135 | 0.8 |
| 5.00 | 125 | 0.82 |
| 5.25 | 100 | 0.84 |

**Imaging on Olympus IX73 Inverted Microscope with Andor Solis Software:**

**Olympus IX73 Microscope:**

1. Turn on camera by flicking switch underneath the metal box.
2. Use the Olympus UPlanSApo 10x/0.40 Objective
3. Turn on LED on the pE-100 for brightfield illumination.
4. For brightfield images, use a filter slot without a filter (intensity 0.6-0.8, exposure: ~0.01 s).
5. Adjust the diaphragm to allow bacteria to be most visible and in focus.
6. For fluorescence images, use filter 6 containing the appropriate filters:	Excitation filter: none; Dichroic mirror: 425 nm; Emission filter: 492 ± 5 nm	Exposure: 0.5 s
7. Use 100% intensity on the UV wavelength on the pE-300^ultra^.
8. Open shutter for fluorescence images to allow LED excitation light to reach sample.

**Imaging with the Andor Solis software:**

1. Login to computer using the password provided.
2. Open the Andor SOLIS software.
3. Press the live image option whilst in brightfield to locate region of interest and focus.
4. Zoom with buttons on the bottom left corner of the image.
5. Single scan for single images, i.e. for brightfield images.
   a. Readout settings:
      i. Pixel Readout Rate: 200 MHz – lowest noise
      ii. Electronic Shuttering Mode: Rolling
      iii. Sensitivity/Dynamic Range: 16-bit (low noise & high well capacity
      iv. Spurious Noise Filter: on
      v. Overlap Readout: off
6. For fluorescence images, use the kinetic series function to capture 45 images at a frequency of 1 (1 image per s).
7. Start shock with the CytePulse device at 15 s into the kinetic series.
8. Save images as .tif files into a unique folder with the following information: voltage, frequency, agarose pad incubation time, and image exposure length.
   a. Settings: 16-bit Gray, 0 – 65535, Full area, Series to Individual Files

**CytePulse Electrode Cleaning Protocol:**

For all washing steps, avoid liquid contact with the IDC port and that only the electrode interface between agarose pads are washed.

8. Prior to cleaning the electrode, ensure cleaning baths are visibly clean and have been changed the same day.
9. Remove agarose pads with rubber coated tweezers or two pipette tips. Be careful not to scratch the electrode surface.
10. Bathe the electrode with the stand in 0.1% Sodium Hypochlorite solution for 10 min.
    a. Prepare by diluting thin bleach in MQ water (e.g. 22 mL 4.53% w/w thin bleach in 978 mL MQ water).

11. Rinse with MQ water with a wash bottle.

12. Place the electrode in an ultrasonic cleaner on the stand containing 0.2% w/v ascorbic acid. Bathe for 7 min, then sonicate for 3 min.
    a. Prepare by dissolving 2.0 g ascorbic acid in 1 L MQ water.

13. Bathe the electrode in MQ water for 10 min.

14. Air dry at room temperature electrode side down on blue roll before storing.

**Image Analysis with Fiji ImageJ:**

6. Open Fiji ImageJ and drag a folder containing the 45 images captured in the kinetic series onto the interface.
7. Click “Yes” when prompted to open the folder as a stack.
8. Adjust the contrast of the stack by selecting a region with bacteria and pressing “Shift + C”, and by selecting “Auto”.
9. Use the function “Plot Z Axis Profile”, found under “Image” > “Stack” > “Plot Z Axis Profile”.
10. A graph of the response of the image stack will be displayed. Press “Live” to update the Z Axis Profile based on the area selected for analysis (i.e. a cluster of cells, or single cells).
